# Toxins Produced by *Valsa mali* var. *mali* and Their Relationship with Pathogenicity

**DOI:** 10.3390/toxins6031139

**Published:** 2014-03-21

**Authors:** Caixia Wang, Chao Li, Baohua Li, Guifang Li, Xiangli Dong, Guoping Wang, Qingming Zhang

**Affiliations:** 1 Key Lab of Integrated Crop Pest Management of Shandong Province, College of Agronomy and Plant Protection, Qingdao Agricultural University, Qingdao 266109, China; E-Mails: cxwang@qau.edu.cn (C.W.); 13864213246@163.com (C.L.); Baohuali@qau.edu.cn (B.L.); liguifangqingnong@163.com (G.L.); xldong@qau.edu.cn (X.D.); 2 College of Plant Science and Technology, Huazhong Agricultural University, Wuhan 430070, China; E-Mail: gpwang@mail.hzau.edu.cn; 3 College of Chemistry and Pharmaceutical Sciences, Qingdao Agricultural University, Qingdao 266109, China

**Keywords:** *Valsa mali* var. *mali*, toxins, HPLC, bioactivity, pathogenicity

## Abstract

*Valsa mali* var. *mali* (*Vmm*), the causal agent of apple tree canker disease, produces various toxic compounds, including protocatechuic acid, *p*-hydroxybenzoic acid, *p*-hydroxyacetophenone, 3-(*p*-hydroxyphenyl)propanoic acid and phloroglucinol. Here, we examined the relationship between toxin production and the pathogenicity of *Vmm* strains and determined their bioactivities in several assays, for further elucidating the pathogenesis mechanisms of *Vmm* and for developing new procedures to control this disease. The toxins were quantified with the high performance liquid chromatography (HPLC) method, and the results showed that the strain with attenuated virulence produced low levels of toxins with only three to four kinds of compounds being detectable. In contrast, higher amounts of toxins were produced by the more aggressive strain, and all five compounds were detected. This indicated a significant correlation between the pathogenicity of *Vmm* strains and their ability to produce toxins. However, this correlation only existed *in planta*, but not *in vitro*. During the infection of *Vmm*, protocatechuic acid was first detected at three days post inoculation (dpi), and the others at seven or 11 dpi. In addition, all compounds produced noticeable symptoms on host plants at concentrations of 2.5 to 40 mmol/L, with protocatechuic acid being the most effective compound, whereas 3-(*p*-hydroxyphenyl)propanoic acid or *p*-hydroxybenzoic acid were the most active compounds on non-host plants.

## 1. Introduction

Apple tree canker disease, a destructive disease in eastern Asia, especially in China, is caused by *Valsa mali* var. *mali* (anamorph *Cytospora* sp.) (*Vmm*) [1,2]. The pathogen causes elongated cankers on branches and trunks, which could lead to death of the entire tree and even result in the failure of the whole orchard [3]. This disease is now epidemic in many main apple producing areas in China and is a problem of economic significance in apple production [4,5,6]. The official data from the Chinese Modern Agricultural Industry Technology System revealed that in 2008, the incidence of apple tree canker disease across the country was 52.7%, with that in some areas being more than 85% [4]. Similarly, in 2011, 68.20% of apple trees grown in Yantai area, Shandong province, suffered from *Vmm*, and the recurrence rate of old canker reached 60.29% [5]. 

Despite the serious damage of this disease, the pathogenesis mechanisms of *Vmm* are still less understood [6,7]. Plant fungal pathogens often cause characteristic symptoms by producing one or more toxic compounds and cell wall-degrading enzymes, all of which have been implicated as pathogenesis factors [8,9,10,11,12]. Researches by Liu *et al*. and Wang *et al*. [13,14] revealed that both inside and outside of the host, *Vmm* produced pectinase, an enzyme that could decay tomato seedlings. Another study by Chen *et al*. [15] systemically analyzed the enzymes produced by *Vmm* during infection, the result of which found that five kinds of cell wall-degrading enzymes with high activities were secreted and that enzyme activities were significantly correlated with the pathogenicity of *Vmm* strains. In other studies, the toxins produced by *Vmm* were investigated, and five kinds of compounds were identified, including protocatechuic acid, *p*-hydroxybenzoic acid, *p*-hydroxyacetophenone, 3-(*p*-hydroxyphenyl)propanoic acid and phloroglucinol [16,17]. Although roles were assigned to those compounds, their bioactivities have yet to be determined. In addition, it remains unknown what the relationship between the production of toxins and the pathogenicity of *Vmm* is. 

The main objectives of this study include: (a) quantifying the toxins produced by *Vmm* using HPLC; (b) evaluating the production of toxins by *Vmm* strains with different pathogenicity, LXS080601 and LXS081501; (c) investigating the dynamics of toxin production and the lesion sizes on apple detached branches after inoculation with the two strains; and (d) determining the bioactivities of toxins produced by *Vmm* in a variety of assays.

## 2. Results

### 2.1. Quantification of Toxins

An HPLC method was developed for the quantification of protocatechuic acid, *p*-hydroxybenzoic acid, *p*-hydroxyacetophenone, 3-(*p*-hydroxyphenyl)propanoic acid and phloroglucinol. In order to acquire the optimal quantification conditions, HPLC parameters and settings were adjusted for each chemical compound. Under the optimal HPLC conditions, the average recoveries ranged from 88.57% to 95.21% for liquid medium and diseased apple branches, with relative standard deviations of 2.3% to 5.6%. The linearity ranges for five kinds of compounds were from 5.00 to 1280.00 μg/g, with correlation coefficients from 0.998 to 1.000. The limit of quantitation (LOQ) was defined as the minimum fortified level of recovery, which was 0.01 μg/g for both liquid medium and diseased branches. The limit of detection (LOD) was 0.005 μg/g at a signal-to-noise (S/N) ratio of 3:1. 

### 2.2. Comparison of Toxins Produced in Vitro

Both strains used in this study grew well in Fuji and Gala branch extract media, but the mycelial growth of LXS081501 was faster than that of LXS080601. After culturing for 15 days, the fungal dry weights for LXS081501 were 2.21 g/100 mL and 2.16 g/100 mL, respectively, when growing in Fuji and Gala branch extract media. In contrast, the dry weights for LXS080601 were just 1.06 g/100 mL and 1.12 g/100 mL in these two media, respectively. The toxin measurements showed that no compounds were detected in the apple branch extract media inoculated with potato dextrose agar (PDA). *Vmm* strain LXS080601 produced five kinds of compounds in these media; however, LXS081501 only produced four compounds with no 3-(*p*-hydroxyphenyl)propanoic acid being detected (Table 1). In Fuji branch extract medium, LXS0801501 produced 3.91-times more phloroglucinol than LXS080601 (overall average of 96.29 ± 5.02 μmol and 24.62 ± 2.45 μmol per gram of fungal dry weight, respectively); in contrast, LXS080601 produced 1.99-, 2.56- and 2.58-times more protocatechuic acid, *p*-hydroxybenzoic acid and *p*-hydroxyacetophenone than LXS081501, respectively. In both strains, phloroglucinol was the most prominent compound produced in either Fuji or Gala branch extract media.

**Table 1:** Production of toxins by *Valsa mali* strains LXS080601 and LXS081501 in Fuji and Gala branch extract media determined 15 days after inoculation. Different letters in the same line indicated significant differences at *p* < 0.05 by Duncan’s multiple range test. “-” indicates that the compound was not detected.

Toxins	Fuji branch extract medium		Gala branch extract medium	
	(μmol/g of fungal dry weight)		(μmol/g of fungal dry weight)	
	LXS080601	LXS081501	LXS080601	LXS081501
Protocatechuic acid	15.56 ± 0.61 b	7.82 ± 0.75 a	15.08 ± 0.60 b	8.03 ± 0.59 a
*p*-Hydroxybenzoic acid	0.55 ± 0.19 b	0.21 ± 0.03 a	0.51 ± 0.07 b	0.23 ± 0.05 a
*p*-Hydroxyacetophenone	1.45 ± 0.20 b	0.56 ± 0.09 a	1.32 ± 0.07 b	0.58 ± 0.05 a
3-(*p*-Hydroxyphenyl)propanoic acid	6.03 ± 0.65 a	-	5.95 ± 0.41 a	-
Phloroglucinol	24.62 ± 2.45 a	96.29 ± 5.02 b	24.34 ± 95.47a	104.31 ± 7.61 b

### 2.3. Comparison of Toxins Produced in Detached Branches

The incidence of infected apple detached branches at 15 days post inoculation (dpi) was not significantly affected by *Vmm* strains, with the average infection frequency caused by LXS080601 and LXS081501 being 100% and 93.33%, respectively. However, these fungal strains caused significantly (*p* < 0.05) different lesion sizes, the average of which was 7.78 cm^2^ for LXS080601 and 1.56 cm^2^ for LXS081501. In the absence of *Vmm* inoculation, no compounds were detected in apple detached branches. LXS080601 produced all five kinds of compounds focused on in this study, but LXS081501 only produced three kinds with no *p*-hydroxyacetophenone and phloroglucinol being detected (Table 2). In addition, given a specific compound, the amount produced by LXS080601 was higher than that by LXS081501. Specifically, LXS080601 produced an average of 1.66-times more protocatechuic acid, 6.57-times more 3-(*p*-hydroxyphenyl)propanoic acid and, especially, about 10-times more *p*-hydroxybenzoic acid than LXS081501. In apple detached branch assays, the pathogenicity of *Vmm* strains was responsible for the diseased lesion sizes, which, interestingly, were also correlated with the quantity and kinds of compounds produced by *Vmm* strains. These results indicated that toxin production was related to the pathogenicity of *Vmm* strains.

**Table 2:** Production of toxins by *Valsa mali* strains LXS080601 and LXS081501 in Fuji and Gala detached branches determined 15 days after inoculation. Different letters in the same line indicated significant differences at *p* < 0.05 by Duncan’s multiple range test. “-” indicates that the compound was not detected.

Toxins	Fuji detached branch		Gala detached branch	
	(nmol/g of tissue dry weight)		(nmol/g of tissue dry weight)	
	LXS080601	LXS081501	LXS080601	LXS081501
Protocatechuic acid	73.71 ± 6.62 b	43.02 ± 3.11 a	70.79 ± 5.90 b	43.84 ± 7.07 a
*p*-Hydroxybenzoic acid	111.35 ± 7.96 b	10.93 ± 1.81 a	97.16 ± 21.94 b	9.63 ± 1.30 a
*p*-Hydroxyacetophenone	43.11 ± 1.91 a	-	38.63 ± 3.97 a	-
3-(*p*-Hydroxyphenyl)propanoic acid	5293.13 ± 166.76 b	864.60 ± 69.93 a	5158.45 ± 147.26 b	735.15 ± 58.86 a
Phloroglucinol	77.95 ± 5.28 a	-	65.03 ± 8.57 a	-

### 2.4. Temporal Dynamics of Lesion Sizes and Toxins after Inoculation

**Figure 1:**
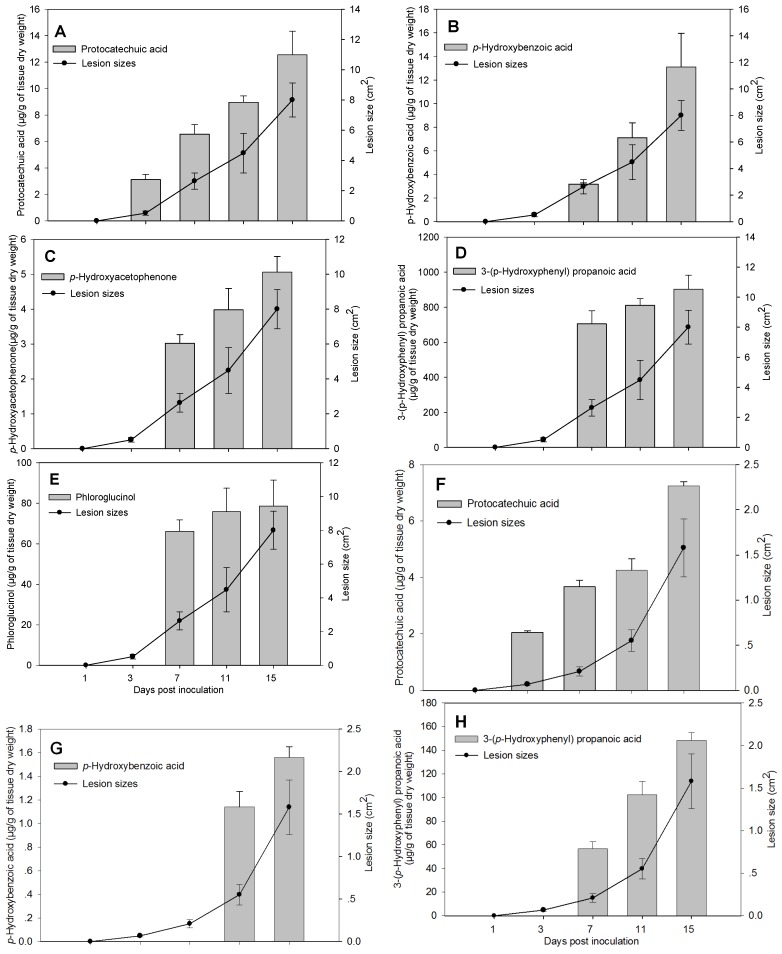
Dynamics of toxins (column) and lesion size (point) in apple detached branches after inoculation with strains LXS080601 (**A** to **E**) and LXS081501 (**F** to **H**). Each column and point represents an average of 20 values (Fuji and Gala cultivars, five replicates, two experiments), and error bars indicate the standard error. The effect of *Valsa mali* strains on all the toxins was significant at *p* < 0.05.

In both apple cultivars, LXS080601 produced more toxins in terms of quantity, as well as categories than LXS081501. As indicated by a significant interaction between the diseased lesion sizes and days post inoculation (*p* < 0.05), the dynamics of toxin accumulation also differed for the two strains (Figure 1). In the apple detached branches inoculated with *Vmm* strain LXS080601, protocatechuic acid was first detected at 3 dpi (3.12 ± 0.41 μg/g of tissue dry weight), and its level significantly increased over time until 15 dpi (Figure 1A), which was accompanied by aggressive lesion expansion. This revealed that there was a significant curved linear relationship between the amount of protocatechuic acid and the corresponding lesion sizes (correlation coefficient, *r* = 0.97). The other four kinds of compounds produced by strain LXS080601 were all first detected at 7 dpi, and their amounts all increased greatly over time (Figure 1B–D), except for phloroglucinol, whose level slightly climbed (Figure 1E). In addition, the correlation coefficients between toxin amount and corresponding lesion sizes were 0.99, 0.94 and 0.88 for *p*-hydroxybenzoic acid, *p*-hydroxyacetophenone and 3-(*p*-hydroxyphenyl)propanoic acid, respectively. 

In the apple detached branches inoculated with *Vmm* strain LXS081501, protocatechuic acid was also first detected at 3 dpi (2.05 ± 0.06 μg/g of tissue dry weight) and underwent a remarkable increase over time (Figure 1F). The lesion size caused by LXS081501 also expanded, and the correlation coefficient was 0.92 between the amount of protocatechuic acid and lesion sizes. In addition, 3-(*p*-hydroxyphenyl)propanoic acid and *p*-hydroxybenzoic acid were first detected at 7 dpi and 11 dpi, and their levels also rose significantly (Figure 1G,H). However, the total amounts of toxins produced by LXS081501 were significantly lower than those by LXS080601.

### 2.5. Bioactivities of Toxins

#### 2.5.1. Toxic Effects of Compounds on Apple Leaves, Callus and Tree Bark

**Figure 2:**
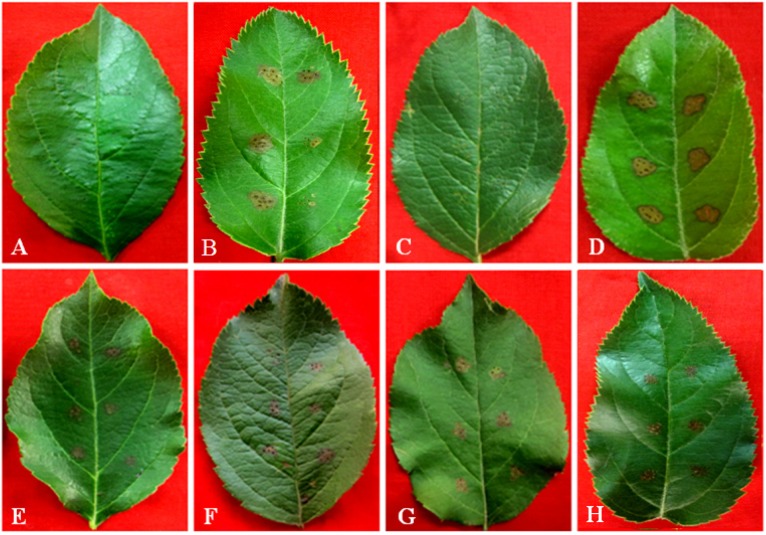
The toxic effects of five kinds of compounds produced by *Vmm* on Fuji leaves. potato dextrose agar (PDA) (**A**) and 20% methanol (v/v) (**C**) show no symptoms, while LXS080601 (**B**); protocatechuic acid (**D**); *p*-hydroxybenzoic acid (**E**); *p*-hydroxyacetophenone (**F**); 3-(*p*-hydroxyphenyl)propanoic acid (**G**); and phloroglucinol (**H**) produce browning necrosis lesions with different sizes. The concentration of phloroglucinol is 40 mmol/L, and for the others is 20 mmol/L.

Since the toxin effects had been attributed to crude *Vmm* extracts [17], it was crucial to test the effect of these compounds on plant tissues in a variety of assays. All these five kinds of compounds produced by *Vmm*, as reported before, were tested with the leaf puncture method. The symptoms that developed within 12 to 24 h depended on the apple cultivars and test compounds. In no case were symptoms observed in 20% (v/v) methanol-and PDA-treated controls. The summary of the bio-activity of test compounds is given in Table 3. At 40 mmol/L, they were all capable of causing brownish necrosis lesions. However, the most bioactive compound was protocatechuic acid, which induced large necrosis lesions with partial falling off of tissues from Fuji leaves. These symptoms appeared to be very similar to those caused by *Vmm* strain LXS080601 on apple leaves (Figure 2A). On Gala leaves, protocatechuic acid caused only brownish necrosis lesions with similar sizes compared to those on Fuji, but no tissues fell off. Overall, Fuji appeared to be more sensitive to these compounds than Gala, which was consistent with the field observations of Abe *et al*. [18,19]. In contrast to the other compounds that all produced large lesions (3 mm × 3 mm to 6 mm × 6 mm) at 20 mmol/L, phloroglucinol did not produce noticeable lesions on apple leaves at the same concentration (Figure 2 and Table 3). 

**Table 3:** Effects of the toxins from *Valsa mali* var. *mali* (*Vmm*) in the leaf puncture wound test on Fuji and Gala. * Lesion size, +++: >6 mm × 6 mm; ++: 3 mm × 3 mm to 6 mm × 6 mm; +: 1 mm × 1 mm to 3 mm × 3 mm; −: <1 × 1 mm. ^#^ Necrotic lesion with partial falling off of tissues.

Toxins	Concentration (mmol/L)	Lesion size *	
		Fuji	Gala
Protocatechuic acid	2.50	+	−
	5.00	++	+
	10.00	++	++
	20.00	+++	++
	40.00	+++^#^	+++
	60.00	+++^#^	+++
*p*-Hydroxybenzoic acid	2.50	−	−
	5.00	+	−
	10.00	+	+
	20.00	++	++
	40.00	++	++
	60.00	+++	+++
*p*-Hydroxyacetophenone	2.50	−	−
	5.00	−	−
	10.00	+	−
	20.00	++	++
	40.00	++	++
	60.00	+++	+++
3-(*p*-Hydroxyphenyl)propanoic acid	2.50	−	−
	5.00	−	−
	10.00	+	+
	20.00	++	++
	40.00	++	++
	60.00	+++	+++
Phloroglucinol	2.50	−	−
	5.00	−	−
	10.00	−	−
	20.00	−	−
	40.00	++	++
	60.00	+++	+++

**Figure 3:**
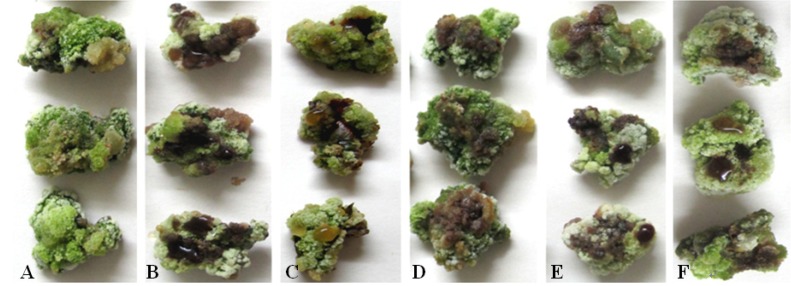
The toxic effects of five kinds of compounds produced by *Vmm* on Fuji calluses. 20% methanol (v/v) (**A**) shows no symptoms, while protocatechuic acid (**B**); *p*-hydroxybenzoic acid (**C**); *p*-hydroxyacetophenone (**D**); 3-(*p*-hydroxyphenyl)propanoic acid (**E**); and phloroglucinol (**F**) produce different degrees of browning around inoculation sites. The concentration of phloroglucinol is 40 mmol/L, and for the others is 20 mmol/L.

**Figure 4:**
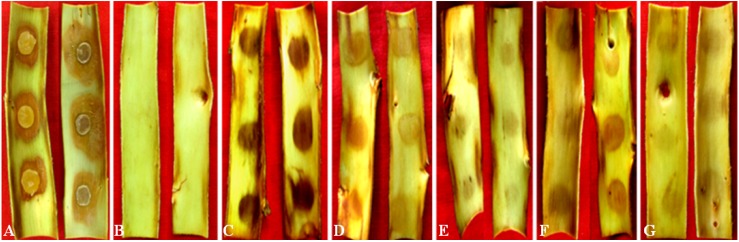
The toxic effects of five kinds of compounds produced by *Vmm* on Fuji tree barks. Strain LXS080601 (**A**) produces the browning lesion around the plugs, while 20% methanol (v/v) (**B**) shows no symptoms. Protocatechuic acid (**C**); *p*-hydroxybenzoic acid (**D**); *p*-hydroxyacetophenone (**E**); 3-(*p*-Hydroxyphenyl)propanoic acid (**F**); and phloroglucinol (**G**) produce browning lesions with distinct colors at the inoculation sites. The concentration of all the compounds is 20 mmol/L.

All the compounds were also tested on Fuji calluses and tree barks. There were no observable symptoms in 20% (v/v) methanol treated controls (Figure 3A and Figure 4B). Within 12 to 24 h, all tested compounds produced obvious symptoms on calluses at 40 mmol/L and on tree barks at 20 mmol/L. As in the apple leaf puncture test, protocatechuic acid was significantly more effective than any of the other compounds (Figure 3 and Figure 4). The least effective compound in these two assays was still phloroglucinol, which necessitated at least 40 mmol/L to cause pronounced symptoms on calluses (Figure 3F). In addition, phloroglucinol produced much slighter browning on tree barks (Figure 4G). 

#### 2.5.2. Toxic Effects of Compounds on Non-Host Plants

**Figure 5:**
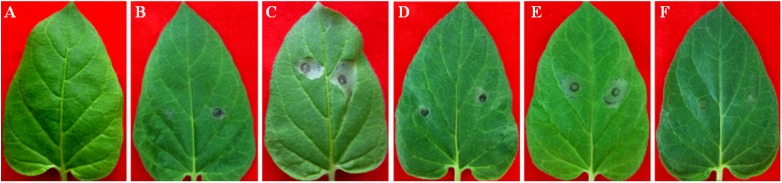
The toxic effects of five kinds of compounds produced by *Vmm* on tobacco leaves. 20% methanol (v/v) (**A**) shows no symptom, while protocatechuic acid (**B**); *p*-hydroxybenzoic acid (**C**); *p*-hydroxyacetophenone (**D**); 3-(*p*-Hydroxyphenyl)propanoic acid (**E**); and phloroglucinol (**F**) produce necrosis lesions in different sizes. The concentrations of all the compounds is 10 mmol/L.

In order to test the bioactivity of all the compounds on non-host plants of *Vmm*, tobacco leaves, as well as tomato, cucumber and Chinese cabbage seeds were selected. All compounds produced noticeable symptoms on tobacco leaves at 10 mmol/L; however, in this assay, *p*-hydroxybenzoic acid was the most effective (Figure 5). These compounds inhibited 50% seed germination at concentrations between 20 and 60 mmol/L. Especially, in the Chinese cabbage germination test, 3-(*p*-hydroxyphenyl)propanoic acid was significantly more effective (20 mmol/L) than any of the other compounds (*p* < 0.05), whereas *p*-hydroxybenzoic acid was the most effective compound (20 mmol/L) in tomato and cucumber germination assays. The least toxic compound in all seed germination assays was phloroglucinol, which required at least 60 mmol/L to inhibit 50% seed germination.

## 3. Discussion

Previous research indicated that the enzymes and toxins produced by fungal pathogens during infection contributed largely to disease development [9,11]. Given defined host-pathogen interactions, specific toxins were employed to promote symptoms [20,21,22]. Therefore, it is of great significance to elucidate the toxin arsenal and its bioactivities, as well as the relationship between toxins and fungal pathogenicity. These studies would lay the solid foundation for investigating the pathogenesis of pathogens and host-pathogen interactions, thus facilitating the exploration of new disease management strategies [23,24,25]. 

Five kinds of compounds produced by *Vmm* were identified by Natsume *et al*. [17]. In this study, we established a rapid, reliable and sensitive HPLC method to detect those compounds. Being able to detect the toxins in apple branch extract medium and infected branches, this method greatly promoted the quantification of toxins produced by *Vmm*. Toxins could also be generated by pathogens cultured on synthetic media, the contents of which impacted the amount of toxins produced [24,25,26,27,28,29]. For example, Czapek medium is most appropriate for *Aristastoma* sp. and *Fusarium oxysporum* to produce toxins, while modified Richard medium is most suitable for *Rhizoctonia solani* [28]. *Vmm* could produce toxins only in apple branch extract medium, but not in Czapek, Richard or PDB medium (PDA without agar), which is consistent with a previous study by Natsume *et al*. [17]. 

In this study, the results from apple branch extract medium and detached branch experiments collectively showed that the *Vmm* strain LXS080601 was able to produce five kinds of compounds; in contrast, LXS081501 could only produce three to four kinds of them. These differences in compound categories were consistent with the pathogenicity of the two strains. While LXS080601 had been reported to infect apple branches and produce larger lesions [15], LXS081501 only caused smaller lesions on branches [30]. With respect to the toxin amount produced by the two *Vmm* strains, in apple branch extract media, LXS081501 produced more phloroglucinol than LXS080601, whereas the latter produced more of the other compounds than the former. In contrast, the quantities of all the compounds produced by LXS080601 were higher than those by LXS081501 in apple detached branches. We examined other 28 *Vmm* strains isolated from different apple production areas in China, and interestingly, the results also revealed a correlation between pathogenicity and toxin production *in planta*, but not *in vitro*. The strains with low toxin production *in planta* were less virulent than those with higher production of toxins. This new and noteworthy finding indicated that the pathogenicity of *Vmm* is highly dependent on its ability to produce toxins *in planta*. 

Numerous studies have compared toxin production *in vitro* and *in planta*. Vogelgsang *et al*. [31] and Reino *et al*. [20] found a correlation between *in vitro* and *in planta* toxin production by *Botrytis* and *Fusarium* species, with similar types of compounds being produced at lower concentrations *in planta*. Our results suggested that there was no significant correlation between *in vitro* and *in planta* toxin production by *Vmm*, since different spectrum and ratios of compounds were produced. This finding was in agreement with the results from Natsume *et al*. [17], who reported that toxin production by *Vmm* *in vitro* were apparently different from those *in planta*. It is well known that all of these five kinds of compounds were the degradation products of phlorizin, a glucoside that is widely distributed in each tissue of the apple tree, including leaves, bark, roots, *etc*. [16,17,32]. Thus, the metabolism of phlorizin by different *Vmm* strains with varying pathogenicity deserves our further study.

It appears that all the compounds were not functioning simultaneously during *Vmm* infection, since, after artificial inoculation, the compounds were first detected at a different time. For instance, protocatechuic acid was first detected at 3 dpi, while the others were at 7 dpi or 11 dpi. This finding indicated that protocatechuic acid displayed a more important role in the establishment of *Vmm* invasion (Figure 1A,F) and that the other toxins may be mainly functional in the process of lesion expansion. In addition, it also suggested a synergistic action of several compounds in the pathogenicity of *Vmm* [17,20]. 

In the current study, the bioactivities of toxins produced by *Vmm* were determined via a series of assays, with each assay having different compounds that were the most active. In the assays on apple leaves, calluses and tree barks, that is, tissues from a host plant of *Vmm*, protocatechuic acid was the most effective compound, which was corroborated by assays on pear (*Pyrus pyrifolia* (Burm.f) Nakai), another host plant of *Vmm* in China. Although toxins produced by *Vmm* are non-host selective [17], in tobacco leaves and seed germination tests of tomato, Chinese cabbage and cucumber (non-host plants), *p*-hydroxybenzoic acid or 3-(*p*-hydroxyphenyl)propanoic acid was the most active. Interestingly, crude toxin preparations, which were the ethyl acetate fraction extracted from diseased apple branches, caused more severe symptoms in apple tissues at 10 mmol/L than a mixture of purified compounds in the same ratios. This finding suggested that additional and yet unknown compounds may also be important for the pathogenicity of *Vmm*. 

## 4. Experimental Section

### 4.1. Fungal Strains, Plant and Medium

The fungal strains used in this study were isolated, identified and maintained as previously described [15,30]. The virulent strain LXS080601 was isolated from Fuji (*Malus domestica* Borkh. cv. Red Fuji), in Qixia, Shandong province, while the strain LXS081501 with attenuated virulence was isolated from *M. spectabilis*, in Urumchi, Xinjiang Uygur Autonomous Region, China. 

Two cultivars of apple, Fuji and Gala (*M. domestica* Borkh.), were used. Both are susceptible to *Vmm*, but Fuji is more likely to be infected than Gala. Branches and leaves for experiments were taken from six-year-grown trees, which were maintained in green house with standard equipment. The Fuji callus was induced and propagated according to the procedure established before [15]. Tobacco seedlings (*Nicotiana glutinous*) were grown in a green house, and the seeds of tomato, Chinese cabbage and cucumber were purchased from the institute of vegetables in Qingdao, Shandong province, China. 

To prepare the apple branch extract medium, 150 g of bark from one to two-year-old Fuji or Gala branches were boiled in water for 30 min. Four layers of gauze were used to filter the solution, to which 40 g of sucrose and 2 g of yeast extract were added. After dissolving, the pH was adjusted to 5.8, and water was added to make the final volume 1 L. Autoclave was performed at 121 °C for 20 min. 

### 4.2. Chemicals

Reference standards of protocatechuic acid, *p*-hydroxybenzoic acid, *p*-hydroxyacetophenone, 3-(*p*-hydroxyphenyl)propanoic acid and phloroglucinol were purchased from Sigma-Aldrich (St. Louis, MO, USA). All chemical solvents were obtained from Beijing Chemical Reagents Company (Beijing, China). All the standard solution stocks of 10 mg/mL were prepared in methanol and stored at −20 °C.

### 4.3. Inoculum Preparation

Plugs (6 mm diameter) were made with a cork borer from the margin of *Vmm* colonies that were grown on potato dextrose agar (PDA) for 3 days at 25 °C in darkness. The plugs were inoculated into barley medium to induce conidia. About one month later, yellow conidia were released and suspended in sterile water to achieve a concentration of 10^6^ conidia/mL. 

### 4.4. Toxin Production in Apple Branch Extract Media

The plugs of strain LXS080601 and LXS081501 were inoculated into the liquid media made with Fuji or Gala branch extract. Six plugs with mycelia were put into each 250-mL flask with 100 mL of medium. The medium inoculated with PDA was used as the control. After culturing for 15 days under 150 rpm/min and 25 °C with a 12-h photoperiod, hypha were collected, dried and weighed. Toxin extraction was performed following a previous protocol [17,33]. Briefly, the pH of the supernatant was adjusted to 3.0 with 1 mol/L of HCl, after which an equal volume of ethyl acetate was added to extract toxins, and the organic phase was saved. This process was repeated three times, and the organic phases were combined. Ethyl acetate was distilled using a rotary evaporator (R220, Buchi, Swiss) under 60 °C, and methanol was used to re-dissolve the residue. The final volume was adjusted to 50 mL, and samples were filtered through a 0.22-μm pore membrane for HPLC analysis. Each experiment was done in triplicate.

### 4.5. Toxin Production in Apple Detached Branches

Healthy branches (one or two years old) with a similar growth status were selected for inoculation experiments. For each 30 to 40 cm branch, four holes with a diameter of 1–2 mm were burnt into each twig segment using an electric iron with an interval hole distance of six to seven cm. Then 50 uL conidia suspension (10^6^ spores/mL) of LXS080601 and LXS081501 was added into each hole, which was then wrapped for 48 h to maintain high humidity. The branches were incubated under 25 °C and 95% relative humidity [7,34]. Branches of diseased areas and margins were collected and crushed into small pieces. Each sample contained 5 g tissues (fresh weight), and then, the samples were freeze-dried and stored at −80 °C for future use. Samples of branches inoculated with water were used as the control. Each experiment was done in triplicate. 

Toxins from diseased branches were extracted according to a method described by Natsume *et al*. [17] with some modifications. Samples were ground to fine powders using a mortar and pestle in liquid nitrogen. Depending on the weight of powders, 1:10 (w/v) pre-cooling 70% methanol was added. The mixture was incubated on ice for 2 h, and the filtrate was collected. This process was repeated once, and the solutions were combined and concentrated in vacuum to a small volume. Then, the residue was diluted with water and extracted with ethyl acetate, after which the obtained toxin compounds were transferred to a 0.05 N NaOH solution and re-extracted with ethyl acetate. The extract was diluted in methanol and subjected to HPLC analysis. 

### 4.6. Toxins HPLC Analysis

The extracts were filtrated through a 0.22-μm pore membrane and then transferred to 2-mL HPLC vials for analysis. A 10 μL sample was analyzed on an Agilent 1100 (Agilent Technologies, Santa Clara, CA, USA) and separated on a 5-μm reversed phase column, C-18, 250 mm × 4.6 mm (Phenomenex, Torrance, CA, USA) maintained at 25 °C. Gradient elution was performed with acetonitrile (Solvent A), methanol (Solvent B) and 0.1% acetic acid solution (C), at a flow rate of 1.0 mL/min. The gradient program to obtain correct separation of the five compounds was: 0–25 min, from 3% A to 15% A and from 97% C to 85% C; 25–35 min, from 15% A to 60% A and from 85% C to 40% C; 35–42 min, keeping 100% B; 42–50 min, 3% A and 97% C. The detection wavelength was 248 nm for phloroglucinol, 260 nm for protocatechuic acid, 254 nm for *p*-hydroxybenzoic acid, 275 nm for *p*-hydroxybenzene propanoic acid and 228 nm for *p*-hydroxyacetophenone, and the corresponding approximate retention times were 5.9 min, 11.8 min, 17.4 min, 25.9 min and 27.0 min, respectively. 

### 4.7. Bioassay of Toxins

#### 4.7.1. Leaves Assay

A simple leaf-puncture assay was used as a rapid guide in determining the toxic effects of toxins [8,35]. The standard chemicals of toxins were diluted from stocks to a series of solutions with different concentrations, and 20 μL from each of them was dripped into an autoclaved Waterman paper disc with a diameter of 6 mm. Fully expanded leaves from Fuji and Gala were collected and pricked by needles to create wounds (six for each leave), onto which the Waterman paper discs containing respective toxin solutions were placed. Inoculated leaves were incubated in a sealed crisper under 25 °C with a 12-h photoperiod. Fuji leaves inoculated with PDA, plugs of strain LXS080601 mycelium grown on PDA or Waterman paper discs containing 20% methanol served as the controls. In each experiment, four leaves were used for each treatment, and this assay was repeated twice.

#### 4.7.2. Callus Test

Fuji calluses with a good growth status were selected and incubated on 2% water agar. Each callus contained five inoculation sites, into each of which 4 μL of toxin solutions were dropped using a pipette. All these inoculated sites had no wounds [15]. Toxin solutions were prepared in the same way as in Section 4.7.1, and the callus inoculated with 20% methanol served as the control. Following incubation in a sealed Petri dish at 25 °C with a 16-h photoperiod, symptoms were carefully examined. Fifteen calluses were used for each treatment, and this experiment was repeated twice.

#### 4.7.3. Tree Bark Assay

The base parts of two-year-old branches were scissored for a length of around 10 cm. Bark was peeled off and the vein cut into two pieces. Each piece of bark contained three inoculating sites with Waterman paper discs containing respective toxin solutions on every site. Bark inoculated with the plugs of strain LXS080601 or Waterman paper discs containing 20% methanol served as the control. Inoculated tissues were incubated in sealed crispers under 25 °C, and symptoms were carefully examined. Four bark tissues were used for each treatment, and this experiment was repeated twice.

#### 4.7.4. Host Selectivity Test

Newly expanded leaves from the upper parts of six- to eight-week-old *N. glutinous* were infiltrated with different concentrations of toxins using a 1-mL syringe without a needle. For each tobacco plant, two leaves were selected with one infiltration (approximate 50 mm^2^) site on each leave. Methanol-infiltrated leaves served as controls. Plants were then grown in a greenhouse under 26–30 °C, and symptoms were carefully examined. Seeds of tomato (*Lycopersicon esculentum* Mill), Chinese cabbage (*Brassica pekinensis*) and cucumber (*Cucumis sativus*) were placed on filter paper impregnated with toxin solutions in a sealed Petri dish that was incubated in darkness at 25 °C. The inhibitions of seed germination were examined [35,36].

### 4.8. Data Analysis

All statistical analysis was performed using the SPSS software standard v. 16.0 (SPSS Inc, Shanghai, China), and the values were expressed as the mean ± the standard deviation (SD). A factorial analysis of variance (ANOVA) was performed to determine the statistical significance (*p* < 0.05) of the main factors and their interactions. 

## 5. Conclusions

The present report has provided new information of great interest about the toxins produced by *Vmm*, because in all cases a positive correlation was demonstrated between the pathogenicity of different strains and their ability to produce toxins in apple branches. This will be useful for further elucidation of the pathogenesis mechanisms of *Vmm* and for better exploration of new strategies to control Valsa canker disease. In addition, protocatechuic acid is the most effective compound on host plants of *Vmm*; however, the most active compounds are different on non-host plants. 
